# Optimizing Nucleic Acid Extraction from Extended Bovine Semen for Endemic and High-Consequence Pathogens

**DOI:** 10.3390/ani15233411

**Published:** 2025-11-26

**Authors:** Amanda Zimmerman, Anne Vandenburg-Carroll, Douglas G. Marthaler, Ailam Lim

**Affiliations:** 1 Wisconsin Veterinary Diagnostic Laboratory, University of Wisconsin-Madison, Madison, WI 53706, USA; aellis7@wisc.edu (A.Z.); vandenburg@wisc.edu (A.V.-C.); 2 Indical Inc., Orlando, FL 32804, USA; douglas.marthaler@indical.com

**Keywords:** semen, extender, extraction, NAHLN, MagMAX CORE, IndiMag Pathogen Kit, influenza A virus, PCR inhibition

## Abstract

Accurate pathogen detection in bovine semen is crucial for animal health surveillance and international trade. Semen presents unique challenges due to the presence of PCR inhibitors from seminal plasma and extender components, reducing nucleic acid extraction efficiency and sensitivity. The two National Animal Health Laboratory Network-approved extraction platforms (MagMAX CORE and IndiMag Pathogen Kits) were evaluated using 88 negative extended semen samples at 200 µL input volume, reduced input volumes, and pretreatment strategies with two influenza A virus (IAV) PCR assays, containing different exogenous internal controls (ICs) to assess PCR inhibition. The ICs yielded overall passing rates from 31.8% to 100.0% and varied greatly based on the extender formulation and extraction protocol. Validation continued with naturally infected semen containing *Mycoplasma bovis*, bovine viral diarrhea virus, bovine herpesvirus-1, and the limit of detection using *Mycoplasma bovis.* The IndiMag Pathogen 100-na was then selected for evaluation of diagnostic sensitivity and specificity, reproducibility, and detection limits with IAV-spiked samples, using the two IAV PCR assays and their ICs. Selected archived semen samples used in this study were screened and were negative for IAV by both PCR assays. These findings underscore the importance of tailored extraction methods in overcoming semen-associated inhibition and facilitating reliable pathogen surveillance in bovine germplasm.

## Simple Summary

Accurate detection of pathogens in bovine semen is vital for animal health surveillance and international trade but is complicated by PCR inhibitors present in seminal plasma and semen extenders. This study compared two extraction platforms using negative extended semen samples at standard (200 µL) and reduced input volumes with different pretreatment approaches. Two influenza A virus (IAV) PCR assays, each with unique exogenous internal controls, were used to evaluate PCR inhibition. Further evaluation with semen naturally infected with *Mycoplasma bovis* (*M. bovis*), bovine viral diarrhea virus, and bovine herpesvirus-1, along with IAV-spiked samples, demonstrates that semen composition strongly influences extraction performance and highlights the importance of optimized extraction protocols to reduce inhibition and enable reliable pathogen detection in bovine germplasm testing.

## 1. Introduction

Screening bovine semen for pathogens is essential for safeguarding animal health, ensuring reproductive success, and maintaining the integrity of national and international breeding programs. Bovine semen can be a vector for transmitting a variety of bacterial, viral, and protozoal pathogens, such as *Brucella abortus*, *Campylobacter fetus*, *Mycoplasma bovis* (*M. bovis*), bovine herpesvirus-1 (BHV-1), bovine viral diarrhea virus (BVDV), and *Tritrichomonas foetus*, especially when proper screening and biosecurity measures are not in place [1,2]. Semen is believed to be the plausible source of the introduction of *M. bovis* into New Zealand [3], illustrating the importance of screening semen as a biosecurity measure.

A foreign animal disease (FAD) in the United States (U.S.), foot and mouth disease virus (FMDV), is detectable and can spread in semen [1]. The potential shed of FMDV in semen could facilitate dissemination both within and between herds, including across geographic boundaries [4,5]. Another FAD, highly pathogenic avian influenza (HPAI) virus, was unexpectedly detected in U.S. dairy cattle, raising concerns about the virus’s capacity for cross-species transmission and spread [6,7]. While respiratory and oral routes remain the primary focus for transmission studies, the detection of HPAI RNA in non-traditional sample types such as raw milk has prompted new questions about alternative pathways for viral spread [7,8]. At present, a single manuscript of HPAI detection in semen from an on-farm breeding bull and semen from a single experimentally HPAI-infected bull has been reported [8,9]. While evidence of natural HPAI infection in bovine semen is lacking and FMDV is absent from the U.S. [10], the topic is important for animal health surveillance and trade requirements.

The detection of pathogen DNA and RNA in semen presents unique technical challenges due to its viscosity and complex biochemical composition. The high concentrations of proteins, nucleases, and polysaccharides, as well as lipid-rich seminal plasma, can interfere with nucleic acid extraction efficiency and downstream amplification, leading to decreased assay sensitivity and the potential for false negatives [1,11,12]. Bovine semen extender is also added to raw bull semen to preserve sperm viability and create multiple doses [13,14]. Extenders contain key components such as egg yolk, milk proteins, soy lecithin, or other plant-based derivatives, which shield the sperm cell membrane from physical and chemical damage, maintain optimal pH (6.8–7.2), and supply metabolizable sugars like fructose or glucose to fuel sperm motility [13,14]. Antibiotics are also included to control bacterial contamination, which can compromise sperm quality and transmit venereal diseases [14]. However, proteins and lipids from extender components can act as PCR inhibitors, potentially complicating nucleic acid extraction from semen and affecting downstream molecular assay performance.

In addition, various pathogens can be intercellular, intracellular, or both, thus requiring a total sample extraction method for pathogen screening [1]. Modified extraction protocols have been published, including the use of centrifugation steps, or the use of reagents capable of mitigating inhibitory effects [4,15,16,17]. Complex and laborious steps prior to the extraction are often required to overcome these numerous issues to obtain high-quality nucleic acid from semen.

The goal of this study was to investigate and develop a viable method to extract nucleic acid from semen with the two National Animal Health Laboratory Network (NAHLN)-approved extraction platforms (MagMAX CORE Nucleic Acid Purification Kit and IndiMag Pathogen Kit) to ensure reliable detection of various pathogens in semen matrices and streamline extraction workflows if semen were part of FAD surveillance.

## 2. Materials and Methods

### 2.1. Field and Reference Samples

Clients routinely submit extended semen samples, consisting of multiple 0.25 cc semen straws, to the Wisconsin Veterinary Diagnostic Laboratory (WVDL) for the surveillance of pathogens by Polymerase Chain Reactions (PCR) assays. Semen samples testing PCR-negative for the pathogens are stored for a minimum of one month, while semen samples testing PCR-positive for pathogens are stored indefinitely for future studies. All subsequently described semen samples were stored in an ultralow freezer at −80 °C until ready for use.

For this study, semen samples testing PCR-negative between January and September 2024 were pooled by submission and extender type consisting of four categories—egg yolk-based extender (Yellow), milk-based extender sexed-semen (Green or Pink), and milk-based extender (White)—to create 88 pooled semen samples. In addition, 36 naturally infected, PCR-positive semen samples, consisting of eight *M. bovis*, five BVDV, and 23 BHV-1 samples submitted between 2008 and 2022 were used.

A single strain of *M. bovis* and three influenza A virus (IAV) reference strains were used for the analytical sensitivity evaluation. The *M. bovis* strain 25523 was acquired from the American Type Culture Collection (ATCC, Manassas VA, USA), while three IAV reference strains were kindly provided by the National Veterinary Service Laboratories (NVSL) Diagnostic Virology Laboratory (Ames, IA) (Appendix A, Table A1). Ten-fold serial dilutions of the references were spiked in White, Yellow, and Green negative semen pools.

Nine HxNx IAV strains (Appendix A, Table A1) at various concentrations were spiked into 27 negative semen pools consisting of White, Yellow, Green, and Pink extenders. These 27 spiked samples, along with 30 additional negative semen pools consisting of the four types of extenders, were used to evaluate diagnostic specificity and sensitivity, repeatability, and the exogenous internal controls (ICs) for the two IAV PCR assays.

### 2.2. Extraction Chemistries and Equipment

The Thermo Fisher Scientific MagMAX CORE Nucleic Acid Purification Kit (MagMAX CORE) (Thermo Fisher Scientific, Waltham, MA, USA) and the INDICAL IndiMag Pathogen Kits (INDICAL BIOSCIENCE, Leipzig, Germany) approved by National Animal Health Laboratory Network (NAHLN) for IAV, African swine fever virus, classical swine fever virus, and FMDV testing were compared, with extractions performed using the Kingfisher Flex (Thermo Fisher Scientific, Waltham, MA, USA) [18,19,20,21].

For the MagMAX CORE extraction evaluation, each of the samples was extracted using the 200 µL sample input volume per semen import requirement by New Zealand Ministry for Primary Industries (CORE 200 with no alteration, CORE 200-na) [22]; a reduced 50 µL sample input volume, similar to the milk testing protocol provided by NAHLN NVSL-SOP-0068 (CORE 50 with no alteration, CORE 50-na) [20]; a reduced 50 µL sample input volume with a pretreatment process using 10 µL of proteinase K, 5 µL of 1M DTT, and 35 µL of 2% SDS, with heating at 60 °C for 5 min (CORE 50-pretreatment); and a reduced 12.5 µL sample input volume with above pretreatment process (CORE 12.5-pretreatment). When the sample input was less than 200 µL, 1× phosphate-buffered saline (PBS) was added to achieve a total input volume of 200 µL (Table A2).

For the IndiMag Pathogen Kit extraction evaluation, samples were extracted using the 200 µL sample input volume, mimicking CORE 200-na for semen import requirement by New Zealand Ministry for Primary Industries (Pathogen 200 with no alteration, Pathogen 200-na); reduced 100 µL sample input volume with 100 µL PBS (Pathogen 100 with no alteration, Pathogen 100-na); and a 100 µL semen with a pretreatment process using 20 µL of proteinase K, 90 µL of Buffer ATL (Qiagen, Germantown, MD, USA), with heating and mixing at 56 °C for 10 min (Pathogen 100-pretreament) (Table A2).

Two microliters of VetMAX Xeno Internal Positive Control RNA (Xeno RNA, Thermo Fisher Scientific, Waltham, MA, USA) and 1 µL of the WVDL internal control (WVDL IC) were added to the lysis solution per sample for each extraction kit. The remainder of the extraction process was conducted according to the manufacturer’s instructions. Eluted RNA was used for PCR evaluation or stored in an ultralow freezer at −80 °C until ready for use.

### 2.3. Polymerase Chain Reactions (PCR)

The RNA extracted using the described methods was evaluated using the NAHLN IAV Matrix PCR assay (NAHLN assay) and the WVDL in-house influenza A Matrix PCR assay (WVDL assay). The NAHLN assay utilized AgPath-ID One Step PCR reagents (Thermo Fisher Scientific, Waltham, MA, USA) and the VetMAX Xeno Internal Positive Control-VIC Assay (Thermo Fisher Scientific, Waltham, MA, USA) for master mix per NVSL-SOP-0068 [20]. The harmonized IAV thermocycling program was used on ABI 7500, with 40 cycles (Thermo Fisher Scientific, Waltham, MA, USA). The NAHLN-approved protocols are available online: https://www.aphis.usda.gov/animal_health/lab_info_services/downloads/ApprovedSOPList.pdf (accessed on 26 September 2025). The NAHLN program office controls the distribution of protocols and the detailed information in the previously listed protocols; these can be requested by emailing NAHLN@usda.gov.

The WVDL IAV, *M. bovis*, BVDV, and BHV-1 assays utilized the VetMAX Fast Multiplex Master Mix (Thermo Fisher Scientific, Waltham, MA, USA). Each assay consisted of primers and probes for the pathogen and the WVDL IC and 5 µL of extracted nucleic acids in a 15 µL reaction. A thermocycling program of 50 °C for 5 min, 95 °C for 10 min, and 40 cycles of 95 °C for 3 s and 58 °C for 30 s was used on ABI 7500 (Thermo Fisher Scientific, Waltham, MA, USA) (Table S1).

### 2.4. PCR and Statistical Analysis

All the PCR analyses were performed at a 5% manual threshold value for the IAV, *M. bovis*, BVDV, BHV-1, Xeno RNA, and WVDL IC to avoid run-to-run fluctuations. A 5% manual threshold setting for Xeno RNA deviated from the auto-threshold setting in the NAHLN NSVL-SOP-0068 [20]. The auto-threshold algorithm was influenced by the samples within each run, resulting in fluctuations between runs. The 5% manual threshold value for pathogen targets and Xeno RNA accurately positioned the threshold around the midpoint of the log-linear phase for the Xeno RNA analysis across all runs.

The effectiveness of the extraction protocol was determined by PCR amplification of the Xeno RNA and WVDL IC. In accordance with the NAHLN acceptance criteria, the passing CT for Xeno RNA and WVDL IC was set at a CT value of <34.5. The various pathogen and internal positive control targets with no amplification were assigned a cycle threshold (CT) value of 40 (the maximum cycle of the assays) for statistical analysis.

The *M. bovis* and IAV assays’ performance was evaluated using the limit of detection (LOD), the coefficient of determination of the standard curve (R^2^), and PCR efficiency. For the LOD, a standard curve was generated using ten-fold dilutions for each of the reference strains that was extracted in triplicate. The endpoint dilution of the reference strain determined the LOD, where all three replicates were detected. PCR efficiency and R^2^ were obtained from the standard curve generated by the Design and Analysis software (Thermo Fisher Scientific, Waltham, MA, USA). The IAV assays were used to evaluate diagnostic sensitivity and diagnostic specificity. Inter-run repeatability and reproducibility were assessed by testing a second set of the same samples four months later.

Paired T-tests and analysis of variance (ANOVA) with multiple comparison tests were used to infer statistical significance among various methods. The statistical analysis was performed using Prism software version 10.6.0 (GraphPad, Boston, MA, USA). Figures were generated using Tableau software, Public Edition (Salesforce, San Francisco, CA, USA).

## 3. Results

### 3.1. Extraction Method Comparison

#### 3.1.1. New Zealand Requirement of 200 µL Semen Input

The first part of the study evaluated the performance of the current NALHN-approved extraction kits, the MagMAX CORE and IndiMag Pathogen kits, using 88 negative extended semen samples with the New Zealand required sample input of 200 µL. The Xeno RNA with the NAHLN IAV matrix PCR as a reference method and the WVDL IC (routinely used to screen semen for pathogens at the WVDL) with the WVDL in-house influenza A PCR assay were utilized to investigate the inhibitory effect. The overall passing rates for the MagMAX CORE with no alteration (CORE 200-na) were 31.8% and 97.7% for Xeno RNA and WVDL IC, respectively. The overall passing rates for the IndiMag Pathogen Kit with no alteration (Pathogen 200-na) were 37.5% and 94.3% for Xeno RNA and WVDL IC, respectively. The passing rate varied significantly (3.1% to 100%) depending on the type of semen extender, ICs, and extraction chemistry (Table 1 and Table S2).

#### 3.1.2. Manufacturer-Suggested Extraction Modifications

The manufacturer-suggested pretreatment protocols were attempted. With MagMAX CORE, a 50 µL input with pretreatment (CORE 50-pretreatment) was attempted, but the elution contained significant bead residue and could not be pipetted due to its clumpiness. Thus, a 12.5 µL input with pretreatment (CORE 12.5-pretreatment) was attempted, along with a 50 µL input with no alteration, similar to the milk testing protocol provided in NAHLN NVSL-SOP-0068 (CORE 50-na). With the IndiMag Pathogen Kit, a 100 µL input with no alteration (Pathogen 100-na) and a 100 µL input with pretreatment (Pathogen 100-pretreatment) were attempted.

**Figure 1:**
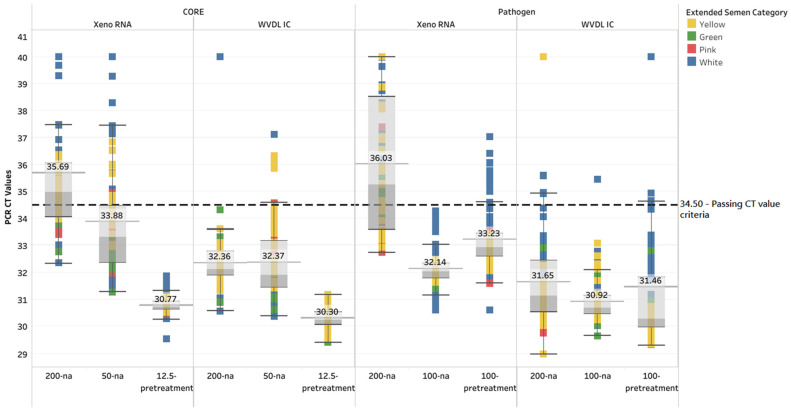
Box and whisker plot illustrating the Xeno RNA and Wisconsin Veterinary Diagnostic Laboratory internal control (WVDL IC) CT values for each semen sample using the MagMAX CORE (CORE) with 200 µL semen input with no alteration (200-na), 50 µL semen input with no alteration (50-na), and 12.5 µL semen input with pretreatment (12.5-pretreatment) and the IndiMag Pathogen Kit (Pathogen) with 200 µL semen input with no alteration (200-na), 100 µL semen input with no alteration (100-na), and 100 µL semen input with pretreatment (100-pretreatment). The categories of semen extenders are egg yolk-based (Yellow), sexed milk-based (Green or Pink), and milk-based (White). The mean CT value is illustrated for each extraction protocol. The passing CT value criteria of 34.50 is represented by the black horizontal dashed line.

Upon testing with the CORE 50-na, overall passing rates increased to 73.9% and 93.2% for the Xeno RNA and the WVDL IC, respectively. The CORE 12.5-pretreatment overall passing rate was 100% for the Xeno RNA and the WVDL IC. For the Pathogen 100-na, the overall passing rate increased to 100% for the Xeno RNA and 98.9% for the WVDL IC. The Pathogen 100-pretreatment reduced the overall passing rates to 88.6% and 85.2% for the Xeno RNA and the WVDL IC, respectively (Table 1). The internal positive control CT values ranged from 29.51 to 40 with the Xeno RNA and from 29.40 to 40 with the WVDL IC. The CT values for the non-passing samples moved into the passing range as successful modifications were applied (Figure 1 and Table S2).

#### 3.1.3. In-Depth Analysis of the PCR Internal Controls

**Figure 2:**
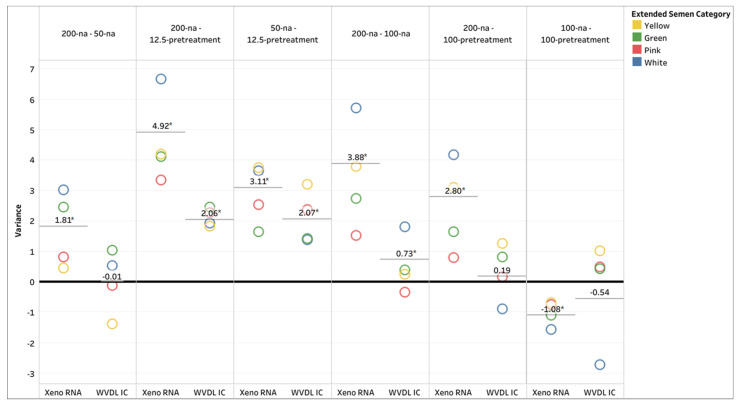
Xeno RNA and Wisconsin Veterinary Diagnostic Laboratory internal control (WVDL IC) CT value variance between the different extraction methods using MagMAX CORE (CORE) with 200 µL semen input with no alteration (200-na), 50 µL semen input with no alteration (50-na), and 12.5 µL semen input with pretreatment (12.5-pretreatment) and the IndiMag Pathogen Kit (Pathogen) with 200 µL semen input with no alteration (200-na), 100 µL semen input with no alteration (100-na), and 100 µL semen input with pretreatment (100-pretreatment). The categories of semen extenders are egg yolk-based (Yellow), sexed milk-based (Green or Pink), and milk-based (White). The asterisks indicate significance with *p* < 0.0001.

For MagMAX CORE, a significant difference in improved CT values was observed from 200-na to 50-na for Xeno RNA only, and from 200-na to 12.5-pretreatment and 50-na to 12.5-pretreatment for both the Xeno RNA and WVDL IC (ANOVA with multiple comparison tests, *p* < 0.0001). The CORE 200-na to 50-na had minimal effect on the mean variance for WVDL IC. The high variance demonstrates successful modification of the protocol for minimizing PCR inhibitory effects (Figure 2 and Table S2).

For the Pathogen Kit, significant improvements in CT values were achieved by reducing the sample input volume with no alteration (Pathogen 200-na to 100-na) for the Xeno RNA and WVDL IC or by incorporating additional pretreatment steps at the reduced volume (Pathogen 200-na to 100-pretreatment) for Xeno RNA only (ANOVA with multiple comparison tests, *p* < 0.0001). The pretreatment provided no significant improvement over the reduced volume samples (Pathogen 100-na to 100-pretreatment). The high variance demonstrates successful modification of the protocol for minimizing PCR inhibitory effects (Figure 2 and Table S2).

### 3.2. Evaluation of Diagnostic Sensitivity for the Selected Extraction Protocols

A total of 36 extended semen samples naturally infected with *M. bovis* (n = 8), BVDV (n = 5), or BHV-1 (n = 23) were extracted in duplicate using separate extractions for the CORE 12.5-pretreatment, Pathogen 100-na, and Pathogen 100-pretreatment protocols with the WVDL pathogen-specific assays (Table 2). The CORE 12.5-pretreatment and the Pathogen 100-na were selected based on the high (98.9–100%) passing rate for both ICs in the first portion of the study; the Pathogen 100-pretreatment was also included to investigate the effect of pretreatment in naturally infected samples. Samples with a pathogen CT value less than 40 were considered positive, regardless of the CT value of the ICs.

**Figure 3:**
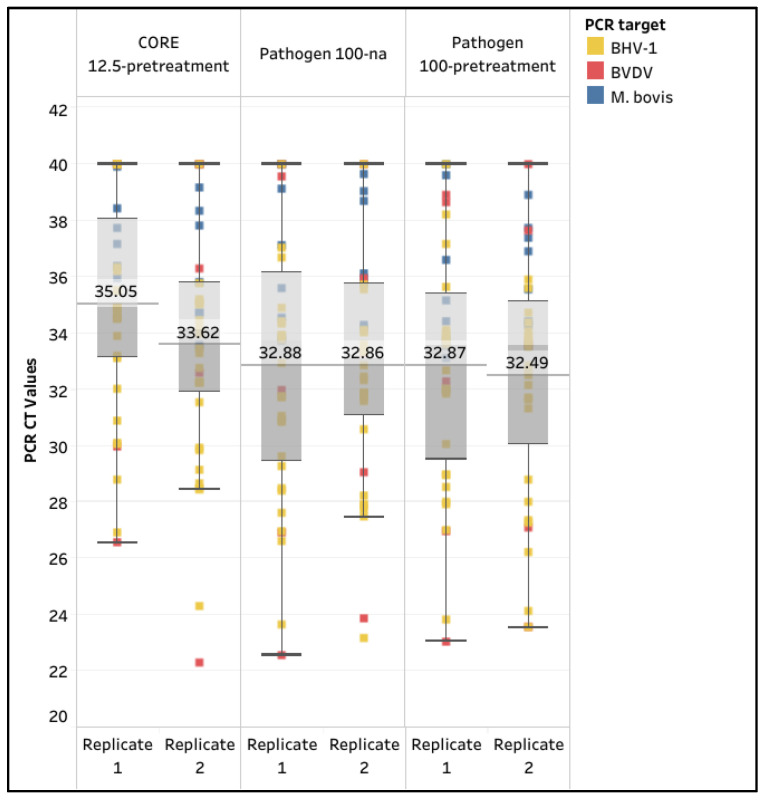
Box and whisker plot illustrating the *Mycoplasma bovis* (*M. bovis*), bovine viral diarrhea virus (BVDV), and bovine herpesvirus-1 (BHV-1) positive extended semen samples with passing internal controls using the MagMAX CORE Kit (CORE) extraction with 12.5 µL semen input and pretreatment (12.5-pretreatment) and using the IndiMag Pathogen Kit (Pathogen) extraction with 100 µL semen input with no alteration (100-na) and 100 µL semen input with pretreatment (100-pretreatment). The mean CT value is illustrated for each extraction protocol.

The diagnostic sensitivity of the CORE 12.5-pretreatment was the lowest, with the replicates at 80.6% and 88.9%, while the Pathogen 100-pretreatment had the highest sensitivity, with the replicates at 94.9% and 97.2%. The overall mean CT values for the CORE 12.5-pretreatment were 35.05 (95% CI: 33.76–36.35) and 33.62 (95% CI: 32.2–35.04), while the Pathogen 100-na CT values were 32.88 (95% CI: 31.29–34.46) and 32.86 (95% CI: 31.45–34.27), and the Pathogen 100-pretreatment CT values were 32.87 (95% CI: 31.41–33.34) and 32.49 (95% CI: 31.05–33.94), respectively. Overall, the Pathogen 100-na and 100-pretreatment protocols provided higher detection rates and lower mean CT values for *M. bovis*, BVDV, and BHV-1, indicating significantly better diagnostic sensitivity compared to the CORE 12.5-pretreatment protocol (ANOVA test, *p* < 0.0001). A lack of significant difference existed between Pathogen 100-na and Pathogen 100-pretreatment protocols (paired T test, *p* = 0.3849) (Figure 3 and Table S3).

### 3.3. Evaluation of Analytical Sensitivity with CORE 12.5-Pretreatment, Pathogen 100-na, and Pathogen 100-Pretreatment for M. bovis

**Figure 4:**
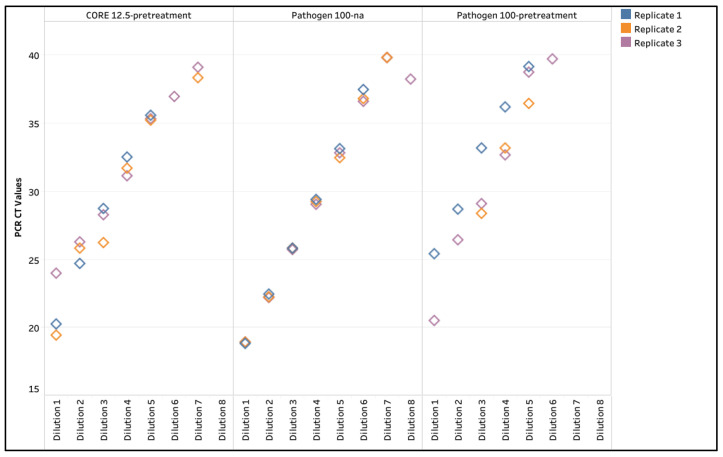
Standard curve for *M. bovis* reference strain *M. bovis* 25523 using the MagMAX CORE with 12.5 μL semen input withpretreatment (CORE 12.5-pretreatment) and the IndiMag Pathogen Kit with 100 μL semen input with no alteration (Pathogen 100-na) and with pretreatment (Pathogen 100-pretreatment).

Understanding the importance of bioexclusion of *M. bovis* for New Zealand, the reference strain *M. bovis* 25523 was diluted ten-fold in prepared negative semen (White) for extraction with CORE 12.5-pretreatment, Pathogen 100-na, and Pathogen 100-pretreatment to evaluate the analytical sensitivity. The LOD was dilution 6 with Pathogen 100-na—with two of the three replicates detected for dilution 7—compared to dilution 5 with CORE 12.5-pretreatment and Pathogen 100-pretreatment (Table 3 and Table S4, Figure 4). The data suggests that the heat treatments were negatively affecting the artificially spiked samples, so these protocols were not further evaluated. For the Pathogen 100-na, the R^2^ values ranged from 0.999 to 1.000, with the percent PCR efficiency ranging between 90.4 and 94.1. The Pathogen 100-na method was selected for subsequent validation in finding with the heat treatment and the simplicity of method.

### 3.4. Evaluation of Pathogen 100-na Protocol with the IAV Assays

#### 3.4.1. Limit of Detection, R^2^, and Percent PCR Efficiency

**Figure A1:**
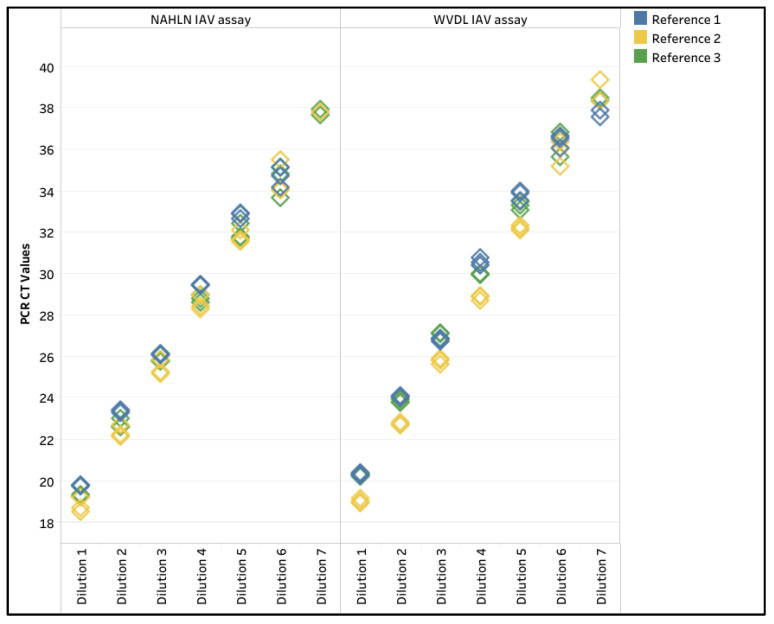
Standard curve for the three influenza A (IAV) reference strains in different extended semen extracted using the IndiMag Pathogen Kit with 100 µL semen input and evaluated on the National Animal Health Laboratory Network (NAHLN) and Wisconsin Veterinary Diagnostic Laboratory (WVDL) IAV assays. Reference 1 was spiked into negative semen with milk-based extender, Reference 2 was spiked into negative semen with egg yolk-based extender, and Reference 3 was spiked into negative sexed-semen with milk-based extender.

Given the epidemic of IAV in U.S. dairy herds and concerns of pathogens in semen, three low pathogenic avian influenza (LPAI) virus reference strains were diluted tenfold in negative semen pools (White, Yellow, and Green). The LOD for the three reference strains was dilution 6 for both the NAHLN and WVDL PCR assays (Table A3 and Table S5, Figure A1). Dilution 7 was detected once or twice out of the three replicates with the NAHLN and WVDL PCR assays (Table A3). The R^2^ values ranged from 0.990 to 0.999, with the percent PCR efficiency ranging between 97.8 and 120.7. The Xeno RNA CT value range in the NAHLN IAV assay was 29.49 to 31.27, while the WVDL IC CT value range in the WVDL IAV assay was 28.87 to 32.14 (Table S5).

#### 3.4.2. Diagnostic Sensitivity and Specificity, and Inter-Run Repeatability

Since the diagnostic sensitivity of naturally infected *M. bovis* samples was previously investigated, and due to limited availability of naturally IAV-infected semen, diagnostic sensitivity and specificity were assessed using 27 IAV-spiked positive and 30 negative semen pools using the NAHLN and WVDL PCR assays, with the same samples extracted four months after the initial extraction for the inter-run repeatability evaluation.

**Figure 5:**
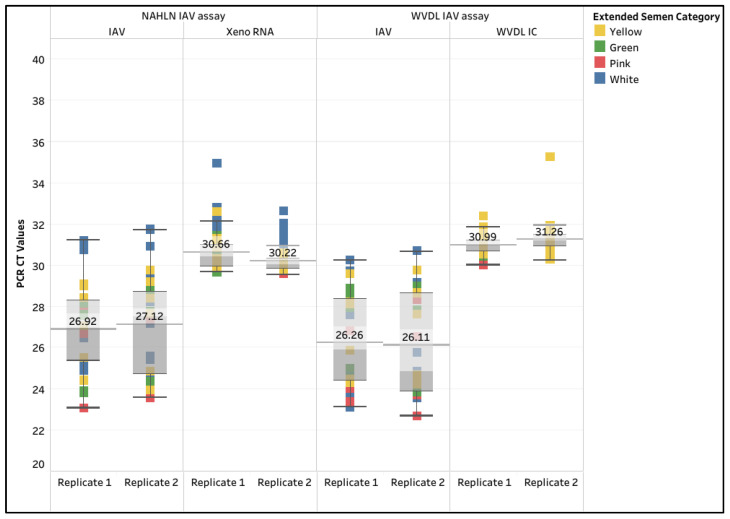
Box and whisker plot of the CT values for the spiked influenza A virus (IAV) and the internal controls in semen extracted using the IndiMag Pathogen Kit with 100 µL semen input on the National Animal Health Laboratory Network (NAHLN) and Wisconsin Veterinary Diagnostic Laboratory (WVDL) IAV assays. The mean CT value is illustrated for each extraction protocol.

A 100% diagnostic sensitivity and specificity occurred with both assays. The mean CT values for IAV using the NAHLN assay were 26.92 (95% CI: 26.04–27.79) and 27.12 (95% CI: 26.20–28.04), while the mean IAV CT values using the WVDL assay were 26.26 (95% CI: 25.37–27.16) and 26.11 (95% CI: 25.14–27.09) (Table S6). For the 57 samples, the overall mean Xeno RNA CT values were 30.66 (95% CI: 30.39–30.92) and 30.22 (95% CI: 30.05–30.39), while the WVDL IC CT values were 30.99 (95% CI: 30.88–31.11) and 31.26 (95% CI: 31.08–31.43), respectively (Figure 5).

**Figure 6:**
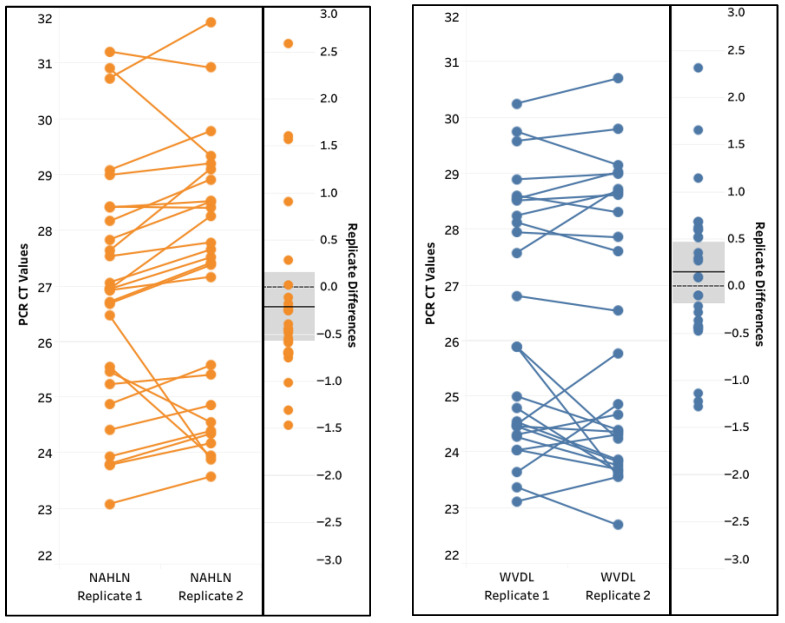
Repeatability for the spiked influenza A virus (IAV) samples extracted using the IndiMag Pathogen Kit with 100 µL semen input on the National Animal Health Laboratory Network (NAHLN) and Wisconsin Veterinary Diagnostic Laboratory (WVDL) IAV assays. The mean variance is illustrated by the solid black line with 95% confidence interval illustrated by the shaded area for each assay.

The inter-run repeatability and reproducibility of the method were assessed through a pairwise comparison of the two sets of replicated testing for the 27 IAV-spiked samples. Overall, excellent correlations were observed among the positive replicates, with Pearson correlation coefficients r = 0.9215 and r = 0.9448 for the NAHLN and WVDL assays, respectively (Figure 6). There was a lack of significant difference between the IAV CT values of the two replicates (Paired *T* test, *p* = 0.2556 and *p* = 0.3557 for the NAHLN and WVDL assays, respectively). The cross-classification of the positive and negative results was 100% with agreement. The data suggests excellent repeatability and reproducibility of the Pathogen 100-na extraction method.

### 3.5. Influenza A Virus Surveillance of Semen Samples Used in This Study

The 88 negative pooled samples for extraction method comparison (Section 3.1) were negative for IAV by the NAHLN and WVDL IAV PCR assays. In addition, the 36 *M. bovis*, BVDV, or BHV-1 naturally infected samples were tested by the NAHLN and WVDL IAV PCR assays to investigate the absences of IAV in historical semen samples. As expected, the samples were negative for IAV. The Xeno RNA CT value range in the NAHLN IAV assay was 27.74 to 31.73, while the WVDL IC CT value range in the WVDL IAV assay was 29.13 to 30.17 (Table S7).

## 4. Discussion

### 4.1. Extraction Method Optimization

Effective isolation of pathogen nucleic acids is necessary in veterinary diagnostics because PCR inhibitors, such as proteins, lipids, and other sample compounds, can generate inaccurate results due to inhibition [23]. In this study, the two NAHLN-approved extraction kits (MagMAX CORE and IndiMag Pathogen) for detecting high-consequence diseases in various sample types, including swabs, tissues, whole blood, and rapidly adapted for IAV surveillance in milk to enable reliable detection and streamlining the laboratory’s surveillance testing, were evaluated [18,19,20,21]. Optimizing the semen extraction protocols for these kits can streamline the detection and surveillance of common pathogens and high consequence diseases, such as FMDV and potentially HPAI, during a U.S. outbreak.

Exogenous ICs are necessary to identify inhibitor effects in the extracted nucleic acid. A previous study using extended semen and the MagMAX CORE protocol with 200 µL input lacked an exogenous IC to monitor PCR inhibition; the semen was diluted in PBS, and assay performance was evaluated based on the detection of spiked *M. bovis* target in only a single semen pool in an unreported extender type [24]. Our study illustrates the variation in inhibitory levels among different samples and different extender types (Figure 1). These factors call into question the validity of using the MagMAX CORE protocol with 200 µL of semen based on the limited data generated in the prior study.

The high occurrence of inhibitors in semen, with disulfide-linked nuclear proteins and lipid-rich seminal plasma, can be addressed by optimizing lysis chemistries using Proteinase K, detergents, reducing agents, and heat treatments to disrupt protein complexes and enhance nucleic acid recovery [25,26,27,28]. In this study, the MagMAX CORE protocols with no alteration (CORE 200-na and 50-na) and the IndiMag Pathogen Kit protocol using 200 µL with no alteration (Pathogen 200-na) did not sufficiently remove inhibition. The CORE 12.5-pretreatment and the Pathogen 100-na sufficiently removed inhibition and drastically improved the passing rate for the Xeno RNA and the WVDL IC. Simply using half of the semen input volume with the IndiMag Pathogen (Pathogen 100-na) reduced inhibition slightly better compared to adding Buffer ATL and heat pretreatment (Pathogen 100-pretreatment).

Since the recommendation of the pretreatment protocols used in this study, the MagMAX CORE protocol was updated in April 2024 to include total semen and 300 µL input with extended heat and proteinase K treatment [29]. Unfortunately, the authors were not notified nor aware of the procedural change. Hence, this study does not compare the MagMAX CORE 300 µL pretreatment protocol to investigate the effect on inhibition removal. However, this protocol was investigated by other researchers and found to be unsuccessful in removing inhibition in extended and raw semen [30].

### 4.2. Evaluation of Diagnostic Sensitivity of the Selected Extraction Protocols

In veterinary diagnostics, careful monitoring of inhibition is critical to avoid reporting of false-negative results, leading to the spread of disease [23,31]. While reducing sample input can minimize PCR inhibition, the lower sample volume may also decrease the sensitivity of pathogen detection. Thus, naturally infected field samples containing *M. bovis*, BVDV, or BHV-1 were tested to verify that the modified CORE (CORE 12.5-pretreatment) and Pathogen (Pathogen 100-na and 100-pretreatment) protocols achieved high sensitivity for pathogen detection, with a slight performance advantage for the Pathogen 100-pretreatment. Similarly, a reduction in milk input volume reduced inhibitors while maintaining sensitivity for IAV surveillance in milk samples [32].

### 4.3. Evaluation of Analytical Sensitivity with CORE 12.5-Pretreatment, Pathogen 100-na, and Pathogen 100-Pretreatment for M. bovis

Due to bioexclusion of *M. bovis* in semen for New Zealand, the reference strain *M. bovis* 25523 was used to evaluate the analytical sensitivity for extraction with CORE 12.5-pretreatment, Pathogen 100-na, and Pathogen 100-pretreatment. Interestingly, the results illustrated that heating negatively impacted the extracellularly spiked target. The Pathogen 100-na illustrated the best analytical sensitivity and is the simpler laboratory method of the three protocols. Subsequently, the Pathogen 100-na protocol was approved as a semen extraction method for New Zealand import requirements (D. Jaramillo, personal communication, 16 October 2025).

### 4.4. Evaluation of Pathogen 100-na Protocol with the IAV Assays

The Pathogen 100-na was further evaluated with spiked IAV semen samples, which achieved high sensitivity for speculative IAV detection and provided the framework for validating this protocol for FMDV detection if a U.S. outbreak were to occur. While all the archived semen samples were negative for IAV (as expected), the recent reports illustrate the potential of finding H5N1 IAV outbreak strains in bull semen through experimental intravenous inoculation or environmental contamination [8,9]. These findings further emphasized the importance of pretreatment strategies and standardized semen nucleic acid extraction protocols to capture both intercellular and intracellular pathogens for surveillance. In addition, the protocol must be simple and high-throughput to remove inhibitors inherent to semen to ensure sensitive and specific detection, limit the spread of endemic and exotic pathogens through germplasm exchange, and support international biosecurity measures [33].

## 5. Conclusions

In conclusion, this study highlights the critical role of optimizing nucleic acid extraction protocols to overcome PCR inhibition in egg-based and milk-based extended semen samples. While reducing sample input volumes and incorporating pretreatments effectively minimized inhibition, a balance must be struck between inhibitor removal and preserving pathogen integrity to maintain high assay sensitivity. Validation with both naturally infected and spiked samples demonstrated that refined extraction protocols could support the reliable detection of viruses and bacteria.

## Figures and Tables

**Table 1:** Percentage of samples passing using the MagMAX CORE Kit (CORE) with 200 µL semen input with no alteration (200-na), 50 µL semen input with no alteration (50-na), and 12.5 µL semen input with pretreatment (12.5-pretreatment); and the IndiMag Pathogen Kit (Pathogen) with 200 µL semen input with no alteration (200-na), 100 µL semen input with no alteration (100-na), and 100 µL semen input with pretreatment (100-pretreatment), using Xeno RNA and Wisconsin Veterinary Diagnostic Laboratory internal control (WVDL IC). The categories of semen are egg yolk-based extender (Yellow), milk-based extender sexed-semen (Green or Pink), and milk-based extender (White).

Extraction Kit	CORE						Pathogen					
Internal Control	Xeno RNA			WVDL IC			Xeno RNA			WVDL IC		
Semen input & modification	200-na	50-na	12.5-pre-treatment	200-na	50-na	12.5-pretreatment	200-na	100-na	100-pretreatment	200-na	100-na	100-pretreatment
Yellow (*n* =24)	25.0	62.5	100.0	100.0	83.3	100.0	41.7	100.0	100.0	95.8	100.0	100.0
Green (*n* = 16)	12.5	100.0	100.0	100.0	100.0	100.0	50.0	100.0	100.0	100.0	100.0	100.0
Pink (*n* = 16)	81.3	87.5	100.0	100.0	93.8	100.0	87.5	100.0	100.0	100.0	100.0	100.0
White (*n* = 32)	21.9	62.5	100.0	93.8	96.9	100.0	3.1	100.0	59.4	83.3	96.9	68.8
Overall % Passing rate (*n* = 88)	31.8	73.9	100.0	97.7	93.2	100.0	37.5	100.0	85.2	94.3	98.9	88.6

**Table 2:** Percentage of pathogen detection for *Mycoplasma bovis* (*M. bovis*), bovine viral diarrhea virus (BVDV), and bovine herpesvirus-1 (BHV-1) positive extended semen samples using the MagMAX CORE Kit (CORE) extraction with 12.5 µL semen input and pretreatment (12.5-pretrement) and using the IndiMag Pathogen Kit (Pathogen) extraction with 100 µL semen input with no alteration (100-na) and 100 µL semen input with pretreatment (100-pretreatment).

	CORE 12.5-Pretreatment		Pathogen 100-na		Pathogen 100-Pretreatment	
Replicate	1	2	1	2	1	2
*M. bovis* (*n* = 8)	75.0%	87.5%	75.0%	100.0%	87.5%	100.0%
BVDV (*n* = 5)	60.0%	60.0%	80.0%	80.0%	100.0%	80.0%
BHV-1 (*n* = 23)	87.0%	95.7%	95.7%	95.7%	95.7%	100.0%
Sensitivity (*n* = 36)	80.6%	88.9%	88.9%	94.4%	94.4%	97.2%

**Table 3:** The limit of detection, coefficient of determination of the standard curve (R^2^), and percent PCR efficiency for *M. bovis* extracted using the MagMAX CORE Kit (CORE) extraction with 12.5 µL semen input and pretreatment (12.5-pretrement) and using the IndiMag Pathogen Kit (Pathogen) extraction with 100 µL semen input with no alteration (100-na) and 100 µL semen input with pretreatment (100-pretreatment).

	CORE 12.5-Pretreatment			Pathogen 100-na			Pathogen 100-Pretreatment		
Replicate	1	2	3	1	2	3	1	2	3
Limit of Detection	5	5	6	6	7	7	5	5	5
R^2^ value	0.996	0.956	0.986	1.000	1.000	0.999	0.994	0.988	0.981
PCR Efficiency (%)	81.6	84.7	133.5	90.4	93.2	94.1	92.8	76.8	71.3

## Data Availability

Raw data can be downloaded at the Supplementary Materials.

## Supplementary Materials

The following supporting information can be downloaded at https://www.mdpi.com/article/10.3390/ani15233411/s1, Table S1. Primers and probes and master mix information for the Influenza A virus (IAV), *Mycoplasma bovis* (*M. bovis*), bovine viral diarrhea virus (BVDV), and bovine herpesvirus-1 (BHV-1) WVDL PCR assays. Table S2: CT values and variance for the NAHLN and the WVDL in-house influenza A virus (IAV) PCR assays for the 88 negative semen samples using the various protocols with the MagMAX CORE (CORE) and the IndiMag Pathogen (Pathogen) kits. Table S3: CT values for *Mycoplasma bovis* (*M. bovis*), bovine viral diarrhea virus (BVDV), and bovine herpesvirus-1 (BHV-1) WVDL PCR assays to assess the extraction method sensitivity for the modified MagMAX CORE (CORE) and IndiMag Pathogen (Pathogen) extraction protocols using 36 naturally infected semen samples. Table S4: CT values for *Mycoplasma bovis* (*M. bovis*) PCR assay to assess the modified MagMAX CORE (CORE 12.5-pretreatment) and IndiMag Pathogen (Pathogen 100-na and Pathogen 100-pretreatment) extraction protocols with the *M. bovis*-spiked samples. Table S5: CT values for NAHLN and WVDL in-house influenza A viurs (IAV) PCR assays with IndiMag Pathogen Kit (Pathogen 100-na) extraction protocol with the IAV-spiked samples. Table S6: CT values for the NAHLN and WVDL in-house influenza A virus (IAV) PCR assays to assess the sensitivity and specificity with IndiMag Pathogen Kit (Pathogen 100-na) extraction protocol with the IAV-spiked samples. Table S7: CT values for the NAHLN and WVDL in-house influenza A virus (IAV) PCR assays to assess the IAV in *Mycoplasma bovis* (*M. bovis*), bovine viral diarrhea virus (BVDV), and bovine herpesvirus-1 (BHV-1) naturally infected semen samples.

## Abbreviations

The following abbreviations are used in this manuscript:

ANOVA	Analysis of Variance
BHV-1	Bovine herpesvirus-1
BVDV	Bovine viral diarrhea virus
CORE	MagMAX CORE Nucleic Acid Purification Kit
CT	Cycle threshold
FMDV	Foot and mouth disease virus
HPAI	Highly Pathogenic Avian Influenza
IAV	Influenza A virus
IC	Internal control
LOD	Limit of detection
LPAI	Low Pathogenic Avian Influenza
MagMAX CORE	MagMAX CORE Nucleic Acid Purification Kit
*M. bovis*	*Mycoplasma bovis*
NAHLN	National Animal Health Laboratory Network
NAHLN assay	NAHLN IAV Matrix PCR assay
NVSL	National Veterinary Service Laboratories
Pathogen	IndiMag Pathogen Kit
PBS	Phosphate-buffered saline
PCR	Polymerase chain reactions
R^2^	Coefficient of determination of the standard curve
Xeno RNA	VetMAX Xeno Internal Positive Control RNA
WVDL	Wisconsin Veterinary Diagnostic Laboratory
WVDL assay	WVDL in-house influenza A Matrix PCR assay
WVDL IC	WVDL internal control

## Appendix A

**Table A1:** List of low pathogenic influenza A viruses used as reference strains and for generation of positive IAV samples for diagnostic sensitivity evaluation.

IAV	Subtype	Strain ID
IAV reference 1	H9N2	Influenza A Virus A/Turkey/CA/6889/1980
IAV reference 2	H5N9	Influenza A Virus A/Turkey/Wisconsin/1968
IAV reference 3	H7N3	Influenza A Virus A/Turkey/Oregon/1977
LPAI 1	H3N8	Influenza A Virus A/Equine/Miami/1/63
LPAI 2	H1N7	Influenza A Virus A/NJ/8/76/EQ-1
LPAI 3	HON3	Influenza A Virus A/NWS-NOV2
LPAI 4	H10N7	Influenza A Virus A/CK/GERM/49
LPAI 5	H2N3	Influenza A Virus A/Mallard/A16/77
LPAI 6	H4N8	Influenza A Virus A/MYNAH/Mass/71
LPAI 7	H7N3	Influenza A Virus A/TY/ORE
LPAI 8	H4N8	Influenza A Virus A/DK/England/62
LPAI 9	H3N8	Influenza A Virus A/DK/Ukraine/1/63

**Table A2:** List of experimental conditions for extraction method evaluation.

Extraction Platform	Protocol	Description of Methodology
MagMAX CORE (CORE)	200-na	200 µL sample
	50-na	50 µL sample and 150 µL PBS
	50-pretreatment	50 µL sample input volume with 10 µL of proteinase K, 5 µL of 1M DTT, and 35 µL of 2% SDS and heat at 60 °C for 5 min. 100 µL of PBS after heating for extraction
	12.5-pretreatment	12.5 µL sample input volume and 35 µL of PBS, with 10 µL of proteinase K, 5 µL of 1M DTT, and 35 µL 2% SDS heat at 60 °C for 5 min. 100 µL of PBS after heating for extraction
IndiMag Pathogen (Pathogen)	200-na	200 µL sample
	100-na	100 µL sample and 100 µL PBS
	100-pretreatment	100 µL semen with 20 µL of proteinase K, 90 µL of Buffer ATL and heating and mixing at 56 °C for 10 min

**Table A3:** The limit of detection, coefficient of determination of the standard curve (R^2^), and percent PCR efficiency for the three reference strains extracted using the IndiMag Pathogen Kit with 100 µL semen input and evaluated on the National Animal Health Laboratory Network (NAHLN) and Wisconsin Veterinary Diagnostic Laboratory (WVDL) assays.

	Reference Strain	NAHLN IAV Assay			WVDL IAV Assay		
		Replicate 1	Replicate 2	Replicate 3	Replicate 1	Replicate 2	Replicate 3
Limit of Detection	1	6	6	6	6	6	6
	2	7	6	6	6	7	7
	3	7	6	7	6	7	7
R^2^ value	1	0.997	0.990	0.994	0.997	0.995	0.999
	2	0.999	0.998	0.998	0.999	0.999	0.996
	3	0.997	0.995	0.999	0.997	0.995	0.996
PCR Efficiency (%)	1	110.6	118.0	113.3	102.1	104.8	103.8
	2	98.5	109.0	101.3	106.2	97.8	101.5
	3	100.6	120.7	110.9	116.4	112.2	109.9
